# Musculocutaneous and median nerve branching: anatomical variations. Case Series from UR clinical anatomy and literature review

**DOI:** 10.4314/ahs.v22i1.33

**Published:** 2022-03

**Authors:** Olivier Kubwimana, Albert Ndata, Andrew Ivang, Paul Ndahimana, Albert Nzayisenga, Jean Claude Byiringiro, Julien Gashegu

**Affiliations:** 1 University of Rwanda, Department of Surgery; 2 Rwanda Military Hospital; 3 University of Rwanda, Clinical Anatomy Unit; 4 Centrede Chirurgie Orthopédique Pédiatriqueetde Réhabilitation Sainte Mariede Rilima

**Keywords:** Anatomical variations, brachial plexus, median nerve, musculocutaneous nerve, upper limb

## Abstract

**Introduction:**

The brachial plexus is highly variable, which is a well-known anatomical fact. Repeated observations on anatomical variations, however, constitute current trends in anatomical research.

**Case series:**

In an anatomical dissection course, three uncommon variations in the brachial plexus were identified in three young adults' cadavers. In one case, the musculocutaneous nerve gave a branch to the median nerve, while the median nerve gave or received musculocutaneous branches in the two remaining corpses.

**Conclusion:**

Anatomical variations of the brachial plexus do occur in our setting. The cases we presented are about anatomical variations of branching patterns of the median and musculocutaneous nerves. Knowledge of those variations is essential for surgery and regional anesthesia of the upper limbs.

## Introduction

The brachial plexus is a network of nerves supplying the motor and sensory innervation to the scapular, pectoral and upper extremity regions. The brachial plexus arises from anterior rami of cervical nerves C5, C6, C7, C8, and T1. Then it passes into the inter- scalenic space between anterior and middle scalene muscles, through the cervico-axillary canal, before entering the axilla and exiting as terminal branches known as peripheral nerves^1^. Though there is a scarcity of information regarding anatomical variations of the brachial plexus in current literature, variations seem to be much more prevalent than one would imagine. With the current increase in surgical procedures of the upper extremities using non-invasive anesthesia^2^, clear understanding of anatomical variations of the brachial plexus is necessary for safe surgical and anesthesia procedures^1^,^2^. We prepared this case series to raise awareness of anatomical variations of the brachial plexus for academic and clinical purposes.

## Case Series

All cases were identified during a structured anatomical dissection course co-organized by the Department of Surgery and the Clinical Anatomy Laboratory of the University of Rwanda for surgical residents, which took place at the Clinical Anatomy Laboratory in Huye Campus on 30^th^ and 31^st^ October 2020. During the practical training, anatomical variations were observed. The course was conducted following the ethical requirements of human cadaveric dissection, and trainees were given clear instructions prior to the procedure. Eight cadavers (16 upper limbs) were dissected. On this sample, we found three cases of musculocutaneous and median nerve anatomical variations.

### Case 1

The first case was found on a 40-year-old male cadaver. The stratigraphic dissection was applied Both pectoralis muscles were reflected laterally, and the brachial plexus was exposed in the axilla. The axillary artery, brachial plexus cords, and their terminal branches were identified. A normal branching of the musculocutaneous and median nerves from the brachial plexus cords was observed. However, while penetrating the coracobrachialis muscle, the right musculocutaneous nerve gave a loop-like communication that anastomosed with the median nerve. This communicating branch was slightly smaller than the musculocutaneous nerve, as illustrated in Figure 1. On further course, the nerve fibers of this anastomotic branch mixed with fibers of the median nerve. However, a distinct bundle of this branch could be seen medially as far as 2 cm above the cubital fossa. From that level it was completely mixed with the fibers of the median nerve.

**Figure:**
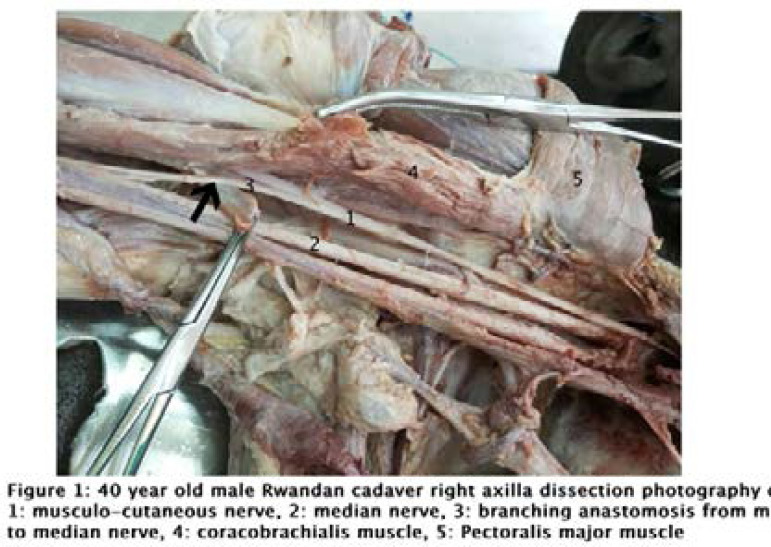


### Case 2

A 20-year-old female cadaver was dissected in the same manner as those in case 1. After the right brachial plexus, the musculocutaneous nerve was found arising from a common trunk with the median nerve. The origin of the musculocutaneous nerve was very distal, closer to the coracobrachialis muscle insertion. During further observation of this branching pattern, it was noted that the lateral cord of the brachial plexus was divided into two separate trunks, which resembled the lateral root of the median nerve (which merged with the medial root of the nerve, forming common nerve steam) and musculocutaneous nerve. However, after a short course, the nerve fibers that resembled the musculocutaneous nerve fused with the fibers of the median nerve. Then, an atypical nerve island was formed at a short distance, and below the island, there was a fusion between the median and musculocutaneous nerve. This fusion resulted in the formation of a common nerve, which we named “median-musculocutaneous nerve,” formed by the lateral root of the lateral cord, seemingly incomplete, and median nerve smaller than expected. The findings are shown in Figure 2.

**Figure:**
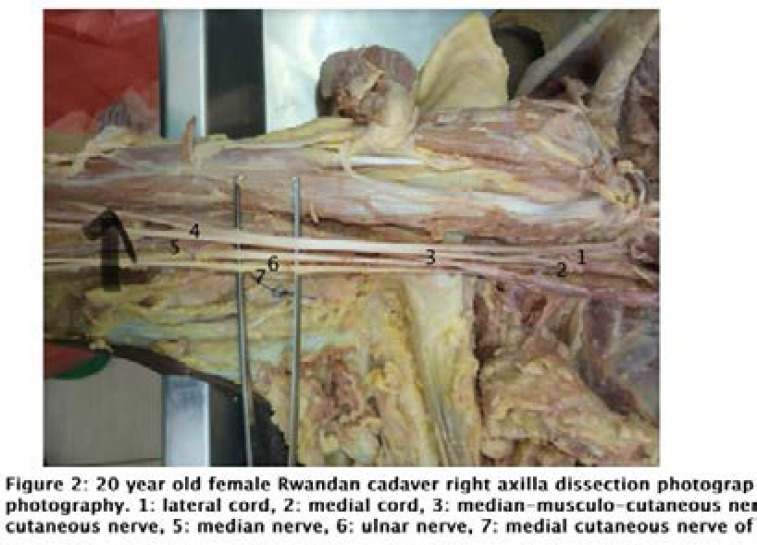


### Case 3

The right axilla of a 24-year-old female cadaver was dissected. in On this specimen, we found the musculocutaneous nerve arising from the median nerve. The origin of the nerve was more proximal in comparison to case 2. The median nerve course was normal. The musculocutaneous nerve did not pierce the coracobrachialis muscle. It ran parallel to the median nerve and gave muscular branches.. From proximal to distal, its branches were: the muscular branches to coracobrachialis and biceps brachii muscles, the lateral cutaneous nerve of the forearm, and the muscular branch to the brachialis muscle. This branching pattern of the musculocutaneous nerve was a wonderfully educational illustration of the function of this nerve. Findings are illustrated in Figure 3.

**Figure:**
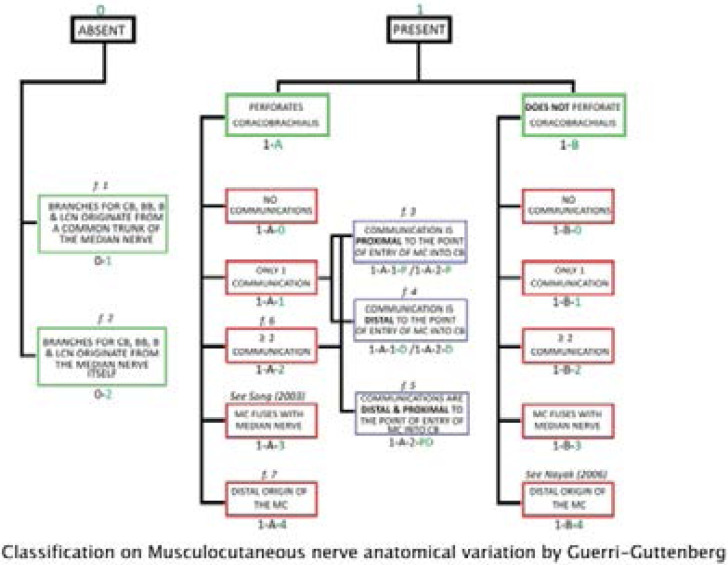


## Discussion

Anatomical variations of the brachial plexus are common and account for 50% of the anatomical variations occurring in the entire nervous system.^1^,^2^ Variations may occur from the origin, where C4 may contribute, resulting in the pre-fixed brachial plexus, or where T2 contributes to the plexus resulting in a post-fixed brachial plexus. Other variations occur in peripheral branches where the musculocutaneous nerve giving a branch to the median nerve, and the median nerve receiving branches from the lateral, medial or posterior cord have been reported.^1^ The musculocutaneous nerve giving a branch to median nerve was also identified by Falougy et al. in the upper part of the arm just below the musculocutaneous nerve where it perforated the coracobrachialis muscle, hence the communication can also be found or seen before the musculocutaneous nerve where it perforates the Coracobrachialis.^3^ The musculocutaneous nerve originating from the median nerve has also been identified previously although some report it has been absent, with an aberrant branch of median nerve. The incidence of musculocutaneous nerve and median nerve communication was reported to be 25% by Caetano et al. in their study on fetal cadaver dissection, and mainly a branch which originates from the musculocutaneous nerve toward the median nerve.^4^ The median nerve and musculocutaneous nerve anatomical variations had been classified by different authors^5^,^6^,^7^ Choi. (?) Guerri-Guttenberg classification is a tentative harmonization of those various classifications. 8 Adegbenro et al., through their cadaveric dissection identified a case of abnormal communicating branch connecting the posterior cord with medial cord compressing on subscapular artery. Moreover, in the same cadaver, the medial cord split proximally, just below its formation and this may bring in various clinical implications.^9^

Anatomical variations of the brachial plexus are common. This is especially true regarding the median nerve and musculocutaneous nerves formation^10^,^11^,^12^,^13^. A partial or complete fusion between the median and musculocutaneous nerves may occasionally be observed^11^,^12^ Median nerve roots may be elongated and may merge into one common trunk outside the axillary cavity, within the arm10. Atypical communications may also exist between the musculocutaneous and median nerves. Moreover, the coexistence of anatomical variations of neurovascular structures may be observed and should be considered during surgical procedures^10^,^11^.

Anatomy dissection is part of clinical anatomy and can enlighten clinical practice as each of the cases described can have particular clinical implications. These cases were quite similar to the previously published cases by the fact that all variations concerned the median nerve and musculocutaneous nerve; moreover they were matching the classification adopted by Guerri-Guttenberg et al.^8^

The first case was classified as 1-A-1-P according to the Guerri-Guttenberg classification because of the musculocutaneous nerve with a communicating branch to the median nerve where it's pierced the coracobrachialis muscle. In cases of entrapment of such musculocutaneous nerve due to spasm of the coracobrachialis muscle, the clinical feature may be challenging as it may combine signs of musculocutaneous nerve injury, weak flexion of the elbow and anesthesia at the lateral aspect of the forearm; and signs of median nerve injury, weak flexion of the wrist or fingers.

The second case was classified 1-A-4 because the musculocutaneous nerve originated from a common trunk with the median nerve and pierced the coracobrachialis muscle. In such case, the musculocutaneous nerve may suffer from possible stretching while performing the Latarjet surgical procedure. The risk of the musculocutaneous nerve being stretched may occur while mobilizing the cut coracoid process around with the coracobrachialis and biceps brevis common tendon. The musculocutaneous nerve is most likely at risk of being stretched in cases where it pierces the coracobrachialis muscle proximally.

The third case was classified 1-B-4 as the musculocutaneous nerve originates distally from a common nerve with the median nerve and had not pierced the coracobrachialis. In this case, the musculocutaneous nerve was at low risk of being stretched while performing the Latarjet surgical procedure. However this anatomical pattern may present an additional risk while exploring the medial groove of the arm in vascular surgery as the musculocutaneous nerve is not a usually expected structure in that area. This case offers an excellent teaching opportunity of the function of the musculocutaneous nerve.

Our two last cases with a distal branching of the musculocutaneous nerve from a common nerve with the median nerve present a favorable pattern for the axillary approach of the brachial plexus blockade as in the usual pattern, the musculocutaneous nerve is hardly blocked as it emerges much higher in the axilla and is therefore not blocked by an injection through this approach.2 The present findings may be reflected to the general population, in that brachial plexus anatomical variation is not as rare as one would imagine. The local/regional awareness of potential human anatomical variation is still low and this may be multifactorial in origin, where culture and other socio-economic factors come in. The clinical anatomy laboratory currently is fostering this practice mainly for undergraduate medical students and surgical trainees in General Surgery, Orthopedics, Plastic surgery, Urology and Neurosurgery.

Repeated observations on anatomical variations constitute current trends in anatomical research. Reports of different types of anatomical variations deepen existing knowledge, help overcome the subjective aspect in the description made by individual researchers, and can be useful for clinicians in their daily practice.^14^

## Conclusion

Anatomical variations of the brachial plexus are not a rare finding and variations may have important clinical implications. Our results revealed that anatomical variations of both musculocutaneous and median nerves described in the literature also exist in the Rwandan population. Further research on these variations are necessary especially in settings like ours where they seem to be more prevalent, to determine whether the common approaches in the shoulder, axilla and arm are safe enough in our population or if they need to be revisited. Surgeons and anesthesiologists operating on the upper extremity in general and brachial plexus in particular should be aware of these variations.
